# Validation of automated data abstraction for SCCM discovery VIRUS COVID-19 registry: practical EHR export pathways (VIRUS-PEEP)

**DOI:** 10.3389/fmed.2023.1089087

**Published:** 2023-10-04

**Authors:** Diana J. Valencia Morales, Vikas Bansal, Smith F. Heavner, Janna C. Castro, Mayank Sharma, Aysun Tekin, Marija Bogojevic, Simon Zec, Nikhil Sharma, Rodrigo Cartin-Ceba, Rahul S. Nanchal, Devang K. Sanghavi, Abigail T. La Nou, Syed A. Khan, Katherine A. Belden, Jen-Ting Chen, Roman R. Melamed, Imran A. Sayed, Ronald A. Reilkoff, Vitaly Herasevich, Juan Pablo Domecq Garces, Allan J. Walkey, Karen Boman, Vishakha K. Kumar, Rahul Kashyap

**Affiliations:** ^1^Division of Critical Care Medicine, Department of Anesthesiology and Perioperative Care, Mayo Clinic, Rochester, MN, United States; ^2^Division of Nephrology and Critical Care Medicine, Department of Internal Medicine, Mayo Clinic, Rochester, MN, United States; ^3^CURE Drug Repurposing Collaboratory, Critical Path Institute, Tucson, AZ, United States; ^4^Department of Information Technology, Mayo Clinic, Scottsdale, AZ, United States; ^5^Division of Critical Care Medicine, Department of Pulmonary Medicine, Mayo Clinic, Scottsdale, AZ, United States; ^6^Division of Pulmonary and Critical Care Medicine, Department of Internal Medicine, Medical College of Wisconsin, Milwaukee, WI, United States; ^7^Department of Critical Care Medicine, Mayo Clinic Florida, Jacksonville, FL, United States; ^8^Department of Critical Care Medicine, Mayo Clinic Health System, Eau Claire, WI, United States; ^9^Department of Critical Care Medicine, Mayo Clinic Health System, Mankato, MN, United States; ^10^Division of Infectious Diseases, Sidney Kimmel Medical College at Thomas Jefferson University, Philadelphia, PA, United States; ^11^Division of Critical Care Medicine, Department of Internal Medicine, Montefiore Medical Center, Albert Einstein College of Medicine, Bronx, NY, United States; ^12^Department of Critical Care Medicine, Abbott Northwestern Hospital, Allina Health, Minneapolis, MN, United States; ^13^Department of Pediatrics, Children’s Hospital of Colorado, University of Colorado Anschutz Medical Campus, Colorado Springs, CO, United States; ^14^Division of Pulmonary, Allergy, Critical Care and Sleep Medicine, Department of Internal Medicine, University of Minnesota Medical School, Edina, MN, United States; ^15^Division of Pulmonary, Allergy, Critical Care and Sleep Medicine, Department of Medicine, Evans Center of Implementation and Improvement Sciences, Boston University School of Medicine, Boston, MA, United States; ^16^Society of Critical Care Medicine, Mount Prospect, IL, United States

**Keywords:** validation, data automation, electronic health records, COVID-19, VIRUS COVID-19 registry

## Abstract

**Background:**

The gold standard for gathering data from electronic health records (EHR) has been manual data extraction; however, this requires vast resources and personnel. Automation of this process reduces resource burdens and expands research opportunities.

**Objective:**

This study aimed to determine the feasibility and reliability of automated data extraction in a large registry of adult COVID-19 patients.

**Materials and methods:**

This observational study included data from sites participating in the SCCM Discovery VIRUS COVID-19 registry. Important demographic, comorbidity, and outcome variables were chosen for manual and automated extraction for the feasibility dataset. We quantified the degree of agreement with Cohen’s kappa statistics for categorical variables. The sensitivity and specificity were also assessed. Correlations for continuous variables were assessed with Pearson’s correlation coefficient and Bland–Altman plots. The strength of agreement was defined as almost perfect (0.81–1.00), substantial (0.61–0.80), and moderate (0.41–0.60) based on kappa statistics. Pearson correlations were classified as trivial (0.00–0.30), low (0.30–0.50), moderate (0.50–0.70), high (0.70–0.90), and extremely high (0.90–1.00).

**Measurements and main results:**

The cohort included 652 patients from 11 sites. The agreement between manual and automated extraction for categorical variables was almost perfect in 13 (72.2%) variables (Race, Ethnicity, Sex, Coronary Artery Disease, Hypertension, Congestive Heart Failure, Asthma, Diabetes Mellitus, ICU admission rate, IMV rate, HFNC rate, ICU and Hospital Discharge Status), and substantial in five (27.8%) (COPD, CKD, Dyslipidemia/Hyperlipidemia, NIMV, and ECMO rate). The correlations were extremely high in three (42.9%) variables (age, weight, and hospital LOS) and high in four (57.1%) of the continuous variables (Height, Days to ICU admission, ICU LOS, and IMV days). The average sensitivity and specificity for the categorical data were 90.7 and 96.9%.

**Conclusion and relevance:**

Our study confirms the feasibility and validity of an automated process to gather data from the EHR.

## Introduction

The pandemic of the coronavirus disease 2019 (COVID-19) has created a need to develop research resources rapidly (1). In response to the global demand for robust multicenter clinical data regarding patient care and outcomes, the Society of Critical Care Medicine (SCCM) Discovery Viral Infection and Respiratory Illness Universal Study (VIRUS) COVID-19 registry was created early in the pandemic (2–4).

Due to the surging nature of pandemic waves, and the subsequent workload and staffing burdens, clinical researchers have encountered difficulty in engaging in rapid, reliable manual data extraction from the electronic health record (EHR) (5). Manual chart review is the gold standard method for gathering data for retrospective research studies (6, 7). This process, however, is time consuming and necessitates personnel resources not widely available at all institutions (8, 9). Prior to the pandemic, automated data extraction from the EHR utilizing direct database queries was shown to be faster and less error-pone than manual data extraction (8, 10). Nonetheless, data quality challenges related to high complexity or fragmentation of data across many EHR systems make automated extraction vulnerable (11–14). Both manual and automatic extraction rely on the EHR, which is an artifact with its own biases, mistakes, and subjectivity (15–20).

Although previous research has looked at these notions, the best methods for obtaining data from EHR systems for research still need to be discovered. In response, we sought to assess the feasibility, reliability, and validity of an automated data extraction process using data for the VIRUS COVID-19 registry.

## Methods

### VIRUS COVID-19 registry

The SCCM Discovery VIRUS COVID-19 registry (Clinical Trials registration number: NCT04323787) is a multicenter, international database with over 80,000 patients from 306 health sites across 28 countries (21). VIRUS COVID-19 registry is an ongoing prospective observational study that aims at real-time data gathering and analytics with a feedback loop to disseminate treatment and outcome knowledge to improve COVID-19 patient care (3). The Mayo Clinic Institutional Review Board authorized the SCCM Discovery VIRUS COVID-19 registry as exempt on March 23, 2020 (IRB number: 20–002610). No informed consent was deemed necessary for the study subjects. The procedures were followed in accordance with the Helsinki Declaration of 2013 (22). Among the participating sites, 30 individual centers are collaborating to rapidly develop tools and resources to optimize EHR data collection. These efforts are led by the VIRUS Practical EHR Export Pathways group (VIRUS-PEEP).

### Data collection

The VIRUS COVID-19 registry has over 500 variables which represents the pandemic registry common data standards for critically ill patients adapted from the World Health Organization- International Severe Acute Respiratory and Emerging Infection Consortium (WHO-ISARIC) COVID-19 CRF v1.3 24 February 2020 (23). The VIRUS-PEEP validation cohort was developed in an iterative, consensus process by a group of VIRUS: COVID-19 registry primary investigators to explore the feasibility of an automation process at each site. The Validation cohort variable was internally validated with seven core VIRUS COVID-19 investigators and subsequently validated from VIRUS-PEEP leads site’s principal investigators. Because of the timeline, the cohort could not be externally validated. A purposeful representative sample of the 25 most clinically relevant variables from each category (Baseline demographic and clinical characteristics of patient and ICU and Hospital-related outcomes) were selected and prioritized for this study (4). We focused on demographic data (age, sex, race, ethnicity, height, weight), comorbidities (coronary artery disease (CAD), hypertension (HTN), congestive heart failure (CHF), chronic obstructive pulmonary disease (COPD), asthma, chronic kidney disease (CKD), diabetes mellitus (DM), dyslipidemia/hyperlipidemia), and clinical outcomes (intensive care unit (ICU) admission, days to ICU admission, ICU length of stay (LOS), type to oxygenation requirement, extracorporeal membrane oxygenation (ECMO), ICU discharge status, hospital LOS, and in-hospital mortality).

To avoid data extraction errors, we utilized precise variable definitions [VIRUS COVID-19 registry code book, cases report form (CRF), and Standard Operating Procedure (SOP)], which were already implemented in the registry and during the pilot phase of the automation implementation. Additionally, all manual and automation data extraction personnel were educated regarding the definitions and procedures needed to collect and report the data.

### System description

De-identified data were collected through Research Electronic Data Capture software (REDCap, version 8.11.11, Vanderbilt University, Nashville, Tennessee) at Mayo Clinic, Rochester, MN, United States (24). The REDCap electronic data capture system is a secure, web-based application for research data capture that includes an intuitive interface for validated data entry; audit trails for tracking data manipulation and export procedures; automated export procedures for seamless data downloads to standard statistical packages; and provide a secure platform for importing data from external sources.

### Manual abstraction

The VIRUS PEEP group has implemented a comprehensive process for data extraction, which involves training manual data extractors. These data extractors are trained to identify, abstract, and collect patient data according to the project’s SOP. During a patient’s hospitalization, extractors follow them until discharge, ensuring that all relevant information is collected. The CRF used in this process includes two main sections: demographics and outcomes, composed of categorical and continuous variables. Extractors answer a mix of binary (“yes” or “no”) and checkbox (“check all that apply”) questions in the nominal variable portions of the CRF. They are instructed to avoid free text and use the prespecified units for continuous variables. In any disagreement, a trainer is always available for guidance and correction. It’s important to note that the manual extractors are unaware of the automated data extraction results.

### Automated extraction

A package of sequential query language (SQL) scripts for the “Epic Clarity” database was developed at one institution and shared through the SCCM’s Secure File Transfer Platform (SFTP) with participating sites. A second site offered peer coaching on the development and utility of end-user Epic™ reporting functions and how to adapt and modify the SQL scripts according to their EHR environment and security firewall. Other tools included R-Studio™ scripts, Microsoft Excel™ macros, STATA 16, and REDCap calculators for data quality checks at participating sites before data upload to VIRUS Registry REDCap. These tools were designed to aid in data extraction, data cleaning, and adherence to data quality rules as provided in VIRUS COVID-19 Registry SOPs. Institutions participated in weekly conference calls to discuss challenges and share successes in implementing automated data abstraction; additionally, lessons learned from adapting the SQL scripts and other data quality tools to their EHR environments were shared between individual sites and members of the VIRUS PEEP group.

### Statistical analysis

We summarized continuous variables of manual and automation process data using mean ± SD and calculated mean difference and SE by matched pair analysis. Pearson correlation coefficient (PCCs) and 95% confidence intervals (CI) were generated for continuous data as a measure of inter-class dependability (25). Pearson correlations were classified as trivial (0.00–0.30), low (0.30–0.50), moderate (0.50–0.70), high (0.70–0.90), and extremely high (0.90–1.00) (26). Bland–Altman mean-difference plots for continuous variables were also provided to aid in the understanding of agreement (27).

Percent agreements were determined for the data collected using each of the two extraction techniques in a categorical variable:

$$\frac{\mathrm{Number}\;\mathrm{of}\;\mathrm{patients}\;\mathrm{categorized}\;\mathrm{identically}\;by\;\mathrm{both}\;\mathrm{sources}\;}{\mathrm{Total}\;\mathrm{number}\;\mathrm{of}\;\mathrm{cases}\;\mathrm{examined}\;by\;\mathrm{both}\;\mathrm{sources}}$$

The total number of agreeing outcomes divided by the total number of results is the summary agreement for each variable. For categorical variables we used Cohen’s kappa coefficient (28). We used the scale created by Landis et al. to establish the degree of agreement (29). This scale is divided by almost perfect (ϰ =0.81–1.00), substantial (ϰ = 0.61–0.80), moderate (ϰ = 0.41–0.60), fair (ϰ = 0.21–0.40), slight (ϰ = 0.00–0.20), and poor (ϰ < 0.00). Additionally, the sensitivity and specificity were calculated by comparing the results of the automated data extractions method to the results of manual data extraction method (gold standard). The 95% confidence intervals were calculated using an exact test for proportions. We used JMP statistical software version 16.2 for all data analysis.

## Results

**Figure 1:**
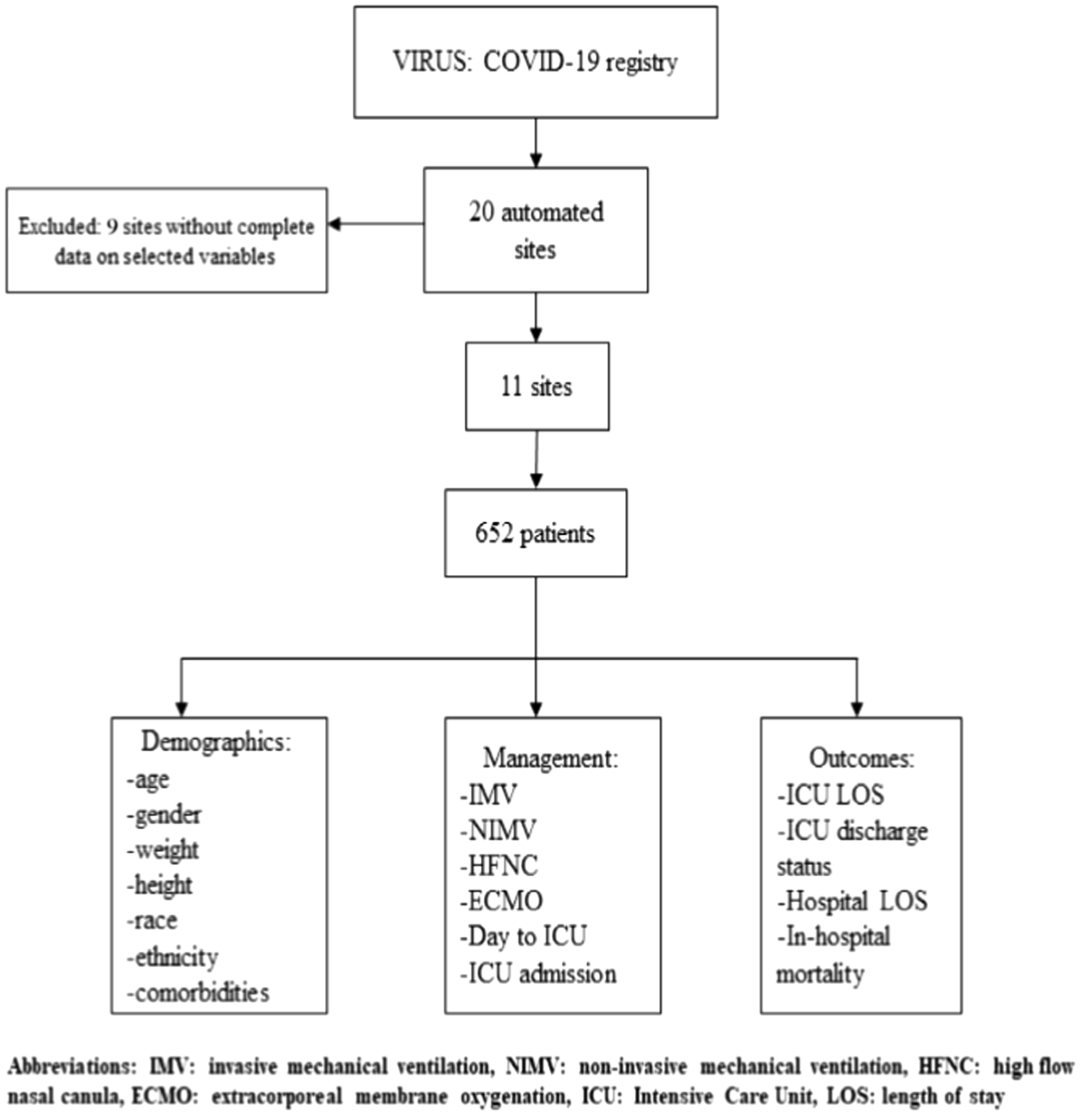
Study flowchart.

Our cohort consisted of data from 652 patients from 11 sites (Figure 1). A total of 25 variables were collected for each patient for manual and automated methods. Of these 25 variables, 16 (64.0%) were nominal, 7 (28.0%) were continuous, and 2 (8.0%) were categorical variables.

**Figure 2:**
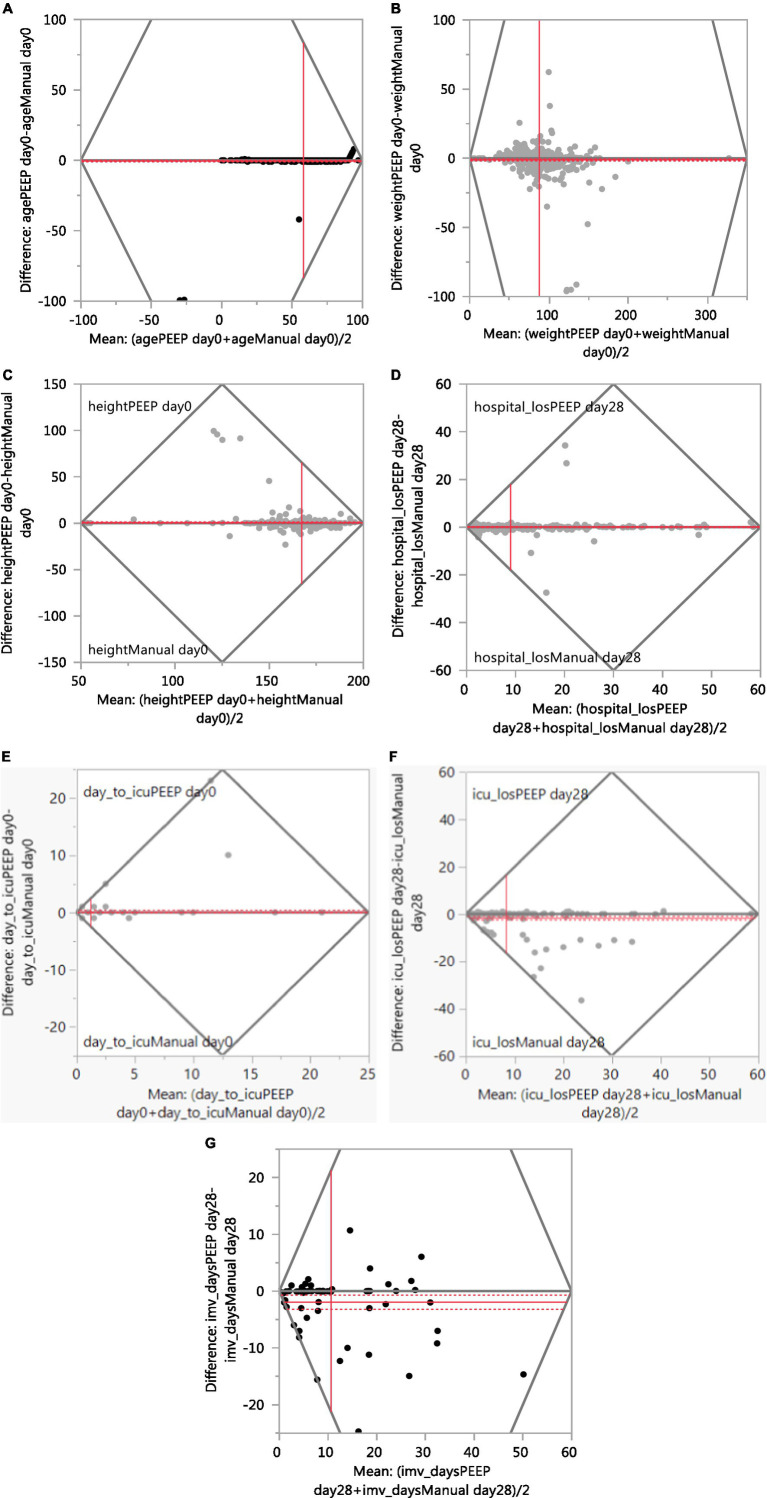
Agreement between manual and PEEP (Bland–Altman plot). **(A)** Age. **(B)** Weight. **(C)** Height. **(D)** Hospital Length of Stay. **(E)** Days to ICU admission. **(F)** ICU Length of Stay. **(G)** IMV Days.

Table 1 summarizes the continuous variables. The automated results for three variables (age, weight and hospital LOS) agreed “extremely high” (>90%) to the manual extraction results. The agreement was “high” (70–90%) for height, days to ICU admission, ICU LOS, and IMV days. Figure 2 presents the Bland–Altman plots for seven continuous variables.

**Table 1:** Comparison of patients in automated versus manual reviews and measures of agreement for individual responses for continuous variables.

Variable name	Automation (Mean, SD)	Manual (Mean, SD)	Mean difference (SE)	Pearson interclass correlation coefficient (PCC), 95% CI	Strength of agreement based on PCC
Age, *N* = 652	57.9 (21.9)	58.5 (19.9)	−0.5 (0.3)	0.95 (0.94–0.96)	Extremely High
Height, *N* = 632	167.6 (15.6)	167 (17.2)	0.6 (0.3)	0.89 (0.87–0.90)	High
Weight, *N* = 632	87.2 (27)	88.4 (28.5)	−1.2 (0.4)	0.94 (0.93–0.95)	Extremely High
Hospital LOS, *N* = 540	9.0 (9.1)	9.0 (9)	0.1 (0.1)	0.97 (0.96–0.97)	Extremely High
Days to ICU admission, *N* = 176	1.3 (3.3)	1.1 (2.6)	0.2 (0.1)	0.80 (0.74–0.85)	High
ICU LOS, *N* = 168	7.5 (9.3)	9.0 (10.5)	−1.5 (0.4)	0.88 (0.85–0.91)	High
IMV Days, *N* = 71	9.7 (9.6)	11.6 (11.1)	−1.9 (0.6)	0.88 (0.81–0.92)	High

CI, Confidence interval; ICU, Intensive Care Unit; IMV, Invasive Mechanical Ventilation; LOS, Length of stay; PCC, Pearson Interclass Correlation Coefficient; SD, Standard deviation; SE, Standard error.

Tables 2, 3 describe the ordinal and nominal variables. The agreement between manual and automated extraction was almost perfect in 13 (72.2%) of the studied variables, and substantial in five (27.8%). The comorbidity “dyslipidemia/hyperlipidemia” had the lowest degree of agreement (moderate 0.61); however, overall percent agreement was high (86.9%). The only variable that showed a Kappa Coefficient equal to 1 was “ICU-discharge status.” The average Kappa Coefficient was 0.81 for the eight comorbidities collected and was 0.86 for outcomes variables, considered almost perfect. The automated electronic search strategy achieved an average sensitivity of 90.7% and a specificity of 96.9%. The sensitivity and specificity of each data-extraction method for all variables are presented in Table 3.

**Table 2:** Comparison of patients in automated versus manual reviews and measures of agreement for individual responses for categorical (ordinal) variables.

Variable name	Automated vs. manual, percent agreement	Kappa coefficient (95% CI, SE)	Strength of agreement based on Kappa coefficient
Race, *N* = 652		0.91 (0.88–0.93, 0.01)	Almost perfect
White Caucasian	365/372 (98.1)		
Black or African American	138/139 (99.3)		
Others	111/141 (78.7)		
Total	614/652 (94.2)		
Ethnicity, *N* = 652		0.88 (0.84–0.93, 0.02)	Almost perfect
Non-Hispanic	506/512 (98.8)		
Hispanic	97/105 (92.4)		
Unknown/Not applicable	23/35 (65.7)		
Total	626/652 (96)		

CI, Confidence interval; SE, Standard error.

**Table 3:** Comparison of patients in automated versus manual reviews and measures of agreement for individual responses for categorical (nominal) variables.

Variable name	Percent agreement, automated vs. manual	Sensitivity	Specificity	Kappa coefficient (95% CI, SE)	Strength of agreement based on Kappa coefficient
Sex, *N* = 652		99.7	99.7	0.99 (0.99–1.0, 0)	Almost perfect
Male	359/360 (99.7)				
Female	291/292 (99.7)				
Total	650/652 (99.7)				
Coronary artery disease, *N* = 540		98.6	97.4	0.90 (0.85–0.96, 0.03)	Almost perfect
Yes	73/74 (98.6)				
No	454/466 (97.4)				
Total	527/540 (97.6)				
Hypertension, *N* = 540		92.0	93.5	0.85 (0.80–0.89, 0.02)	Almost perfect
Yes	298/324 (92.0)				
No	202/216 (93.5)				
Total	500/540 (92.6)				
Congestive heart failure, *N* = 540		88.0	97.8	0.82 (0.74–0.90, 0.04)	Almost perfect
Yes	44/50 (88)				
No	479/490 (97.8)				
Total	523/540 (96.7)				
Chronic obstructive pulmonary disease, *N* = 540		92.7	96.3	0.80 (0.72–0.88, 0.04)	Substantial
Yes	51/55 (92.7)				
No	467/485 (96.3)				
Total	518/540 (95.9)				
Asthma, *N* = 540		93.7	95.8	0.81 (0.73–0.88, 0.04)	Almost perfect
Yes	59/63 (93.7)				
No	457/477 (95.8)				
Total	516/540 (95.6)				
Chronic kidney disease, *N* = 540		81.2	96.2	0.79 (0.72–0.85, 0.03)	Substantial
Yes	95/117 (81.2)				
No	407/423 (96.2)				
Total	502/540 (93)				
Diabetes mellitus, *N* = 540		92.1	96.3	0.89 (0.85–0.93, 0.02)	Almost perfect
Yes	176/191 (92.1)				
No	336/349 (96.3)				
Total	512/540 (94.8)				
Dyslipidemia/Hyperlipidemia, *N* = 540		88.9	86.4	0.61 (0.53–0.69, 0.04)	Substantial
Yes	80/90 (88.9)				
No	389/450 (86.4)				
Total	469/540 (86.9)				
ICU admission rate, *N* = 611		90.3	95.2	0.86 (0.82–0.90, 0.02)	Almost perfect
Yes	215/238 (90.3)				
No	355/373 (95.2)				
Total	570/611 (93.3)				
IMV rate, *N* = 582		87.7	98	0.85 (0.79–0.92, 0.03)	Almost perfect
Yes	64/73 (87.7)				
No	499/509 (98)				
Total	563/582 (96.7)				
NIMV rate, *N* = 581		83.3	99.3	0.80 (0.66–0.95, 0.07)	Substantial
Yes	15/18 (83.3)				
No	559/563 (99.3)				
Total	574/581 (98.3)				
HFNC rate, *N* = 581		100	98.9	0.86 (0.75–0.97, 0.06)	Almost perfect
Yes	19/19 (100)				
No	556/562 (98.9)				
Total	575/581 (99)				
ECMO rate, *N* = 581		72.7	99.3	0.69 (0.47–0.91, 0.11)	Substantial
Yes	8/11 (72.7)				
No	566/570 (99.3)				
Total	574/581 (98.8)				
ICU discharge status, *N* = 172		100	100	1.0 (1–1, 0.0)	Almost perfect
Death	9/9 (100)				
Alive	163/163 (100)				
Total	172/172 (100)				
Hospital discharge status, *N* = 541		90	100	0.94 (0.88–1, 0.03)	Almost perfect
Death	27/30 (90)				
Alive	511/511 (100)				
Total	538/541 (99.4)				

CI, Confidence interval; ECMO, Extracorporeal membrane oxygenation; HFNC, High Flow Nasal Canula; ICU, Intensive Care Unit; IMV, Invasive Mechanical Ventilation; LOS, Length of stay; NIMV; Non-Invasive Mechanical Ventilation; PCC, Pearson Interclass Correlation Coefficient; SE, Standard Error.

## Discussion

The automated search strategy for EHR data extraction was highly feasible and reliable. Our investigation observed substantial and almost perfect agreement between automated and manual data extraction. There was almost perfect agreement in two-thirds of the categorical variables, and all continuous variables showed Extremely High or High agreement.

The results of our validation study are similar to other studies that validated and evaluated automated data (30–33). Singh et al. (31) developed several algorithm queries to identify every component of the Charlson Comorbidity Index and found median sensitivity and specificity of 98–100% and 98–100%, respectively. In the validation cohort, the sensitivity of the automated digital algorithm ranged from 91 to 100%, and the specificity ranged from 98 to 100% compared to ICD-9 codes. These results are comparable to our study as the comorbidities analyzed presented a sensitivity and specificity of 90.2 and 96.8%, respectively. Our results are superior to the results of Schaerfer et al. (34), who found a sensitivity of 72% and a specificity of 95% for comorbidities (CHF, cerebral vascular disease, CKD, cancer, DM, human immunodeficiency virus, HTN) in patients with COVID-19 pneumonia using ICD-10 base-data comparing to manual data collection. We also successfully compared seven continuous variables with three extremely high agreement and four high agreement in comparison to Brazeal et al. (35), who compared two variables (age and BMI) for manual versus automation in a study population comprised of patients with histologically confirmed advanced adenomatous colorectal polyp.

Manual data extractors can overcome diverse interface issues, read and analyze free text, and provide clinical judgment when retrieving and interpreting data; however, manual data extraction is limited to human resources and is prone to human error (7, 32, 36). In addition to requiring considerable amount of time, manual data extraction also necessitates qualified personnel (30, 33). During the COVID-19 pandemic, where real-time data is paramount, automated data has proven validity and efficacy, and may divert personnel to patient care and other vital tasks. Nonetheless, automated data is not flawless. A significant limitation is finding a unique algorithm that can be applied to every center. Variables collected as free text fields are another challenge for such validations. The automated VIRUS COVID-19 sites had reported over a large majority of variables collected using this method. Currently, more than 60,000 patients and their data variables in the registry had been collected through efforts of the VIRUS-PEEP group, which has allowed for updates and complete data in the shortest possible time.

### Challenges in automation

The environment for data collection is often a shared environment within an institution, and there are limitations on how much data may be extracted and processed in one job and how much post-abstraction processing is necessary. Microsoft SQL and TSQL solutions process substantial amounts of data from many different tables and can take a long time to run on large populations. There are clinical documentation differences between the various sites requiring additional coding when applying the data requirements and rules. Establishing logic for data elements within a given EHR can be time consuming up front, requiring close collaboration between clinician and analytics teams. Data may be stored differently between multiple medical centers in one institution, requiring processing to comply with data requirements for standardization. While sites can share coding experience in data abstraction between similar data storage structure, variable coding schemes pose challenges for direct translation between sites. Lastly, one information technology employee often works on such projects with competing priorities.

### Strengths and limitations

To our knowledge this is first multicenter study to evaluate the feasibility of automation process during COVID-19 pandemic. This automation process should be applicable to any EHR vendor (EHR type agnostic), and these purposeful sampled representative data points would be relevant to any other clinical study/trial, which is a major strength of this study. Nonparticipation of 19 sites out of 30 sites in the VIRUS-PEEP group, which leads to a possibility of selection bias, is a major limitation. The time constraints in the ongoing pandemic at participating sites were the reason behind this non-participation in the validation process. However, extracting data across 11 different centers is one of the strengths of this study; it could also highlight the variations in staff, procedures, and patients at these institutions. Although the SQL queries could be applicable in most sites, some sites required a new SQL tailored to their data architecture. One key limitation for our group was that all sites found a portion of data extraction that could not be automated, including variables which are described in narrative, such as, patient symptoms, estimated duration of onset of symptoms, and imaging interpretations. Another limitation is a notable discrepancy between manual and EMR extraction for important outcomes like ICU LOS and IMV days. The automation process relies on procedure order date (intubation/extubation) and ADT (hospital/ICU admission discharge transfer) order date and time and discontinuation date in EHR; however the manual extractor look for first-time documented ICU or IMV in her, which probably could account for such notable discrepancy in outcomes like ICU LOS and IMV days. Transferring a patient to a location that was not a usual ICU due to COVID-19 surge may be another possible explanation for the observed lower sensitivity of ICU admission rate. Variation in creation of make-shift ICUs at different institution may have caused this discrepancy in automation of ICU admissions documentation. It partially explains the lower sensitivity and high specificity of ICU admission, IMV, NIMV, and ECMO rates by automation process. Another noticeable issue was that the manual data extraction was done in real time and automation was done when the patient was discharged and mainly relied on billing codes and manually verified data available in EHR.

### Future direction

Future research on this topic could involve a thorough comparison of all patient records extracted using two methods: manual extraction and automated SQL queries. The data comparison could be done by aligning data points across a wide range of variables for each data extraction method and then statistically analyzing their consistency and discrepancies. This detailed comparison would verify the reliability of automated data extraction and provide insights into areas that could be improved for greater accuracy.

## Conclusion

This study confirms the feasibility, reliability, and validity of an automated process to gather data from the EHR. The use of automated data is comparable to the gold standard. The utilization of automated data extraction provides additional solutions when a rapid and large volume of patient data needs to be extracted.

## Data availability statement

The original contributions presented in the study are included in the article/supplementary material, further inquiries can be directed to the corresponding author.

## Ethics statement

The studies involving humans were approved by Mayo Clinic Institutional Review Board. The studies were conducted in accordance with the local legislation and institutional requirements. The ethics committee/institutional review board waived the requirement of written informed consent for participation from the participants or the participants’ legal guardians/next of kin because The Mayo Clinic Institutional Review Board authorized the SCCM Discovery VIRUS COVID-19 registry as exempt on March 23, 2020 (IRB number: 20–002610). No informed consent was deemed necessary for the study subjects. The procedures were followed in accordance with the Helsinki Declaration of 2013.

## Author contributions

DV and VB contributed equally in the defining the study outline and manuscript writing. VB, SH, JC, MS, AT, MB, SZ, NS, RC-C, RN, DS, AN, SK, KAB, J-TC, RM, IS, RR, and KB did the data review and collection. DV, VB, and SH did the statistical analysis. VH, JD, AW, VK, and RK did the study design and critical review. DV, VB, SH, and RK were guarantor of the manuscript and took responsibility for the integrity of the work as a whole, from inception to published article. All authors contributed to the article and approved the submitted version.

## Abbreviations

CAD: Coronary artery disease
CHF: Congestive heart failure
CI: Confidence interval
CKD: Chronic kidney disease
COPD: Chronic obstructive pulmonary disease
CRF: Case report forms
DM: Diabetes mellitus
ECMO: Extracorporeal membrane oxygenation
EHR: Electronic health records
HFNC: High flow nasal canula
HTN: Hypertension
ICU: Intensive care unit
IMV: Invasive mechanical ventilation
IRB: Institutional review boards
LOS: Length of stay
NIMV: Non-invasive mechanical ventilation
PCC: Pearson interclass correlation coefficient
REDCap: Research electronic data capture software
SCCM: Society of critical care medicine
SD: Standard deviations
SE: Standard error
SFTP: Secure file transfer platform
SOP: Standard operating procedure
SQL: Sequential query language
VIRUS: Viral Infection and Respiratory Illness Universal Study
VIRUS-PEEP: VIRUS Practical EHR Export Pathways group
WHO: World Health Organization
WHO-ISARIC: World Health Organization- International Severe Acute Respiratory And Emerging Infection Consortium
